# An enhanced regimen as post-exposure chemoprophylaxis for leprosy: PEP++

**DOI:** 10.1186/s12879-018-3402-4

**Published:** 2018-10-05

**Authors:** Liesbeth F Mieras, Anna T Taal, Wim H van Brakel, Emmanuelle Cambau, Paul R Saunderson, W Cairns S Smith, Cita Rosita S Prakoeswa, Linda Astari, David M Scollard, Dejair Caitano do Nascimento, Jacques Grosset, Hemanta K Kar, Shinzo Izumi, Laura Gillini, Marcos C L Virmond, Marieke G G Sturkenboom

**Affiliations:** 1 Netherlands Leprosy Relief, Amsterdam, Netherlands; 2 National Reference Center for mycobacteria and antimycobacterial resistance, Paris, France; 3 American Leprosy Missions, Greenville, USA; 4 Institute of Applied Health Sciences, University of Aberdeen, Aberdeen, Scotland UK; 5 Dermatology Health & Venereology, Faculty of Medicine, Airlangga University, Surabaya, Indonesia; 6 National Hansen’s Disease Programs, Baton Rouge, LA USA; 7 Instituto Lauro de Souza Lima, Bauru (SP), Brazil; 8 Johns Hopkins University School of Medicine, Baltimore, USA; 9 Department of Dermatology, Leprosy and STD, Paras Hospitals, Gurgaon, India; 10 Leprosy Study Group, Institute of Tropical Disease, Airlangga University, Surabaya, Indonesia; 11 Global Leprosy Programme, WHO, New Delhi, India; 12 Department of Clinical Pharmacy and Pharmacology, University Medical Center Groningen, Groningen, Netherlands

**Keywords:** Post-exposure prophylaxis, Chemoprophylaxis, Intervention study, Leprosy, Rifampicin, Moxifloxacin, Clarithromycin

## Abstract

The ongoing transmission of *Mycobacterium (M.) leprae* reflected in a very slow decline in leprosy incidence, forces us to be innovative and conduct cutting-edge research. Single dose rifampicin (SDR) as post-exposure prophylaxis (PEP) for contacts of leprosy patients, reduces their risk to develop leprosy by 60%. This is a promising new preventive measure that can be integrated into routine leprosy control programmes, as is being demonstrated in the Leprosy Post-Exposure Programme that is currently ongoing in eight countries.

The limited (60%) effectiveness of SDR is likely due to the fact that some contacts have a preclinical infection beyond the early stages for which SDR is not sufficient to prevent the development of clinical signs and symptoms of leprosy. An enhanced regimen, more potent against a higher load of leprosy bacteria, would increase the effectiveness of this preventive measure significantly.

The Netherlands Leprosy Relief (NLR) is developing a multi-country study aiming to show that breaking the chain of transmission of *M. leprae* is possible, evidenced by a dramatic reduction in incidence. In this study the assessment of the effectiveness of an enhanced prophylactic regimen for leprosy is an important component. To define the so called PEP++ regimen for this intervention study, NLR convened an Expert Meeting that was attended by clinical leprologists, public health experts, pharmacologists, dermatologists and microbiologists.

The Expert Meeting advised on combinations of available drugs, with known efficacy against leprosy, as well as on the duration of the intake, aiming at a risk reduction of 80–90%. To come to a conclusion the Expert Meeting considered the bactericidal, sterilising and bacteriostatic activity of the potential drugs. The criteria used to determine an optimal enhanced regimen were: effectiveness, safety, acceptability, availability, affordability, feasibility and not inducing drug resistance.

The Expert Meeting concluded that the enhanced regimen for the PEP++ study should comprise three standard doses of rifampicin 600 mg (weight adjusted when given to children) plus moxifloxacin 400 mg given at four-weekly intervals. For children and for adults with contraindications for moxifloxacin, moxifloxacin should be replaced by clarithromycin 300 mg (weight adjusted).

## Background

### Introduction

This is a report of an Expert Meeting to propose an enhanced chemoprophylaxis regimen to be tested in a randomised controlled intervention study for contacts of leprosy patients. The meeting was held in November 2016, in Amsterdam, the Netherlands, and was attended by clinical leprologists as well as epidemiologists/public health leprosy experts, pharmacologists, dermatologists and microbiologists. All participants are co-authors of this paper. This report deals only with considerations related to the selection of agents to be used in a prophylaxis study, and does not describe a protocol for the field study. The protocol will be discussed in a future publication.

### Background on post-exposure prophylaxis for leprosy

In the past decade a very slow decline in leprosy incidence and evidence of ongoing transmission of *M. leprae* has been observed. To achieve elimination of leprosy in terms of zero new cases, transmission has to be stopped first. Predictions made, using the individual-based mathematical model SIMCOLEP, show that leprosy is likely to remain a problem in high endemic regions without additional control measures [1]. One of the main challenges is the long incubation period that is around five years on average [2]. Clinical diagnosis is most commonly based on clinical signs and symptoms: a defined skin lesion with loss of sensation, thickened nerves with definite loss of sensation or weakness of hands/feet, presence of visible deformities and/or a positive skin smear. The need for treatment is decided by a clinician [3].

The presence of *M. leprae* in nasal swabs from asymptomatic community members, as determined by PCR or by the presence of antibodies against leprosy bacilli, is dynamic over time [4, 5]. However, existing links between asymptomatic community members testing positive and people with clinical disease seem to suggest the possibility of transmission by subclinical cases [6]. Further evidence is needed to show whether asymptomatic carriers can in fact transmit *M. leprae*. Contacts of people affected by leprosy are known to be at higher risk of developing the disease [7, 8]. Therefore providing post-exposure prophylaxis (PEP) to contacts of leprosy patients may help to stop transmission and will contribute to reducing the number of new leprosy cases. Several chemoprophylactic regimens have been demonstrated to be effective. For example, dapsone given once or twice weekly for at least two years reduced the risk of developing leprosy by 60% and acedapsone, given every ten weeks for seven months, reduced this risk by 49% [9–11]. A single dose of rifampicin (SDR) given to contacts of leprosy patients also showed to reduce the risk of developing leprosy by approximately 60% in the first two years [12]. A combination of rifampicin, ofloxacin and minocycline (ROM) has shown protective efficacy similar to that observed in studies using rifampicin alone, but it was not more bactericidal [13, 14].

PEP with SDR has been adopted as national policy in several countries with a very low leprosy burden, such as Cuba (since 2002), Morocco (since 2014) and Samoa (since 2015). PEP was implemented as a sub-national policy in recent years in Indonesia, Nepal and India [15]. Furthermore, the leprosy post-exposure prophylaxis (LPEP) programme is currently ongoing in eight countries: Tanzania, India, Nepal, Sri Lanka, Myanmar, Brazil, Cambodia and Indonesia. This operational research programme is carried out in partnership with the Ministries of Health and members of the International Federation of Anti-Leprosy Associations (ILEP), with support of the Novartis Foundation. The purpose of the LPEP programme is to learn more about the most effective and acceptable ways of integrating prophylactic treatment into routine leprosy control programmes and to study the impact of SDR on the case detection rates of leprosy in the study districts [16]. Preliminary results show that the level of acceptance of SDR among leprosy patients and their contacts is very high and that the integration of a single dose prophylaxis in routine leprosy control is feasible [17].

However, SDR is only 60% effective in preventing leprosy among contacts of new patients [12]. At least one likely reason for the limited effectiveness of chemoprophylaxis with SDR is that manifestations of the disease cannot be prevented with one single dose of rifampicin in contacts with a preclinical infection beyond the very early stages. This hypothesis is supported by the aforementioned limited effectiveness of SDR, especially in blood-related household contacts in the COLEP trial, among whom SDR only prevented leprosy in less than 30% [12]. Based on scientific reasoning, it is likely that the effectiveness of the current SDR regimen could be increased if contacts of leprosy patients with pre-clinical disease would be treated with an appropriate enhanced prophylactic regimen. A second reason for the limited effectiveness of SDR is the short half-life of rifampicin in the blood (just over 3 h) and the fact that only one dose is administered. Using an antibiotic with a longer half-life in plasma, either on its own or in combination with rifampicin, in repeated doses is likely to enhance the bactericidal effect on *M. leprae.*

### Background on the PEP++ research project

NLR has prepared a study protocol for a multi-country study on stopping the transmission of *M. leprae*, in which the assessment of the effectiveness of an enhanced prophylactic regimen for leprosy is an important component. NLR convened this Expert Meeting to develop a consensus proposal for such an enhanced regimen of which the effectiveness would be compared to SDR as part of this study. The request was to choose a combination of available drugs, with known efficacy against leprosy, and a duration of such an enhanced prophylactic regimen, aiming at a risk reduction of 80–90%, 20–30% more effective than SDR. We call this the PEP++ regimen.

The PEP++ research project will test the enhanced regimen using a cluster-randomised trial design to compare the effectiveness of the enhanced post-exposure prophylaxis regimen with that of SDR in close contacts (household contacts, neighbours and social contacts) who have a higher risk of developing leprosy [8]. Contacts will be followed-up after taking SDR or PEP++ to determine the difference in case detection rate in both groups. Besides an assessment of the effectiveness of the regimen, the PEP++ research project will include a perception of leprosy study, an acceptability study and a cost-benefit analysis. The research protocol will be described in detail in a separate publication.

In short, the PEP++ research project aims to provide evidence that a combination of a large-scale application of the current SDR and PEP++ will be able to prevent the development of leprosy disease among contacts and, as a consequence, stop the transmission of *M. leprae* in previously high-endemic areas. A novel cluster-based blanket implementation of SDR will be used, including all inhabitants of an area in which a cluster of leprosy patients is identified. Clusters in the participating districts will be identified using geographic information system (GIS) technology. The interventions will be supported by optimised leprosy case detection and treatment services, including but not limited to health systems strengthening, contextualised community education on leprosy, stigma reduction interventions and involvement of leprosy-affected persons in various roles in their communities.

## Main text

### Possible drugs for enhanced chemoprophylaxis regimen

The mechanism of chemoprophylaxis is either killing of the causative organisms before the onset of pathology or killing of the pathogen allowing sub-clinical disease to heal or both. In leprosy, because the incubation period is very long, both mechanisms may be involved, though the latter may be more likely. The ideal enhanced regimen would reduce the risk of developing leprosy considerably more than 60% without inducing selection of drug-resistant mutants in leprosy, or other infectious diseases such as tuberculosis, which would lead to a higher risk of relapse and transmission of drug-resistant leprosy.

The Expert Meeting agreed that the PEP++ regimen should be comprised of drugs that are available in leprosy endemic countries and are approved by the United States Food and Drug Administration (FDA) and the European Medicines Agency (EMA). Bedaquiline, oxazolidinone and nitro-dihydro-imidazo-oxazoles were excluded, because they have not been registered in the countries where the PEP++ research project is planned to take place (India, Brazil and Indonesia) or have not been used for the treatment of leprosy. The standard multidrug therapy (MDT) for leprosy, introduced in 1982, is a combination of dapsone and rifampicin for paucibacillary (PB) leprosy, and dapsone, rifampicin and clofazimine for multibacillary (MB) leprosy. MDT is associated with very low relapse rates in patients who complete treatment [18]. Other drugs with known efficacy against leprosy are rifapentine, fluoroquinolones (such as ofloxacin, levofloxacin and moxifloxacin), minocycline and clarithromycin [19].

In order to select the most potent drugs for the regimen, the Expert Meeting considered the bactericidal activity (ability to kill replicating bacteria), the sterilising activity (ability to kill non-replicating bacteria – persisters) as well as the bacteriostatic activity (ability to prevent bacterial growth) of the potential drugs. The effectiveness of the drugs discussed below has been evaluated on established infections in mouse footpads.

**Rifampicin**, routinely used for the treatment of leprosy, blocks the RNA-polymerase which is the first step of protein synthesis and prevents the replication of *M. leprae* without killing on the spot actively-replicating bacteria. However, blocking the synthesis of proteins that are involved in the basic metabolism of non-replicating bacteria (persisters) prevents their survival, which means that rifampicin is actually a key sterilising drug. Shepard et al. [20] demonstrated a complete loss of infectivity in mice of inocula from five lepromatous patients treated for 7 days with a daily dose of 600 mg of rifampicin. Rifampicin is well tolerated and when used as a single or repeated monthly dose very few side effects are seen, apart from discoloration of urine [21].

**Rifapentine** is a rifamycin derivative like rifampicin, but has a longer plasma half-life of 14 to 18 h compared to rifampicin with only 3 h. The killing power of *M. leprae* with a single dose rifapentine, determined by the proportional bactericidal technique in the mouse footpad model [22], was 99.6% in contrast to the bactericidal activity of rifampicin which was 92.1% [23]. Also, *M. leprae* shows a lower minimum inhibitory concentration (MIC) for rifapentine than for rifampicin [24]. Furthermore, fewer doses are needed to prevent relapse (12 weeks) in mouse models of tuberculosis, compared to 24 weeks for rifampicin, because the sterilising effect of rifapentine is stronger [25]. However, the newly developed molecular viability assays indicate that the bactericidal effect of rifamycin derivates may be less rapid than the mouse footpad methods suggested [26]. In conclusion, rifapentine would be a good choice as chemoprophylactic drug for leprosy because of its strong anti-mycobacterial potential. Nevertheless, experience with rifapentine in leprosy patients is limited and it is currently unavailable in most countries. It is accepted by the FDA for use in TB in the USA, but not by the EMA in Europe.

Even though the working mechanism of **clofazimine** against *M. leprae* is not fully understood, it appears to involve interference with the proton motive force and therefore bacterial adenosine triphosphate (ATP) production by membrane interaction with the respiratory chain and/or phospholipids [27]. Clofazimine would be a potential candidate for the PEP++ regimen, because it is already used in MB MDT. Nevertheless, it has two big disadvantages: it lacks early bactericidal activity, taking weeks of daily use to build an effective tissue concentration, and it causes discoloration of the skin after about one month of treatment. Especially, the latter is a major concern for some people, because it is noticed by others and can take a long time to disappear. It is not known whether giving clofazimine intermittently is as effective as a daily dose, nor is it known whether intermittent use would completely prevent skin discoloration.

**Dapsone** is a bacteriostatic drug, used for leprosy treatment since 1945. It inhibits the bacterial nucleic acid synthesis, the building blocks of bacterial DNA. The infectiousness of *M. leprae*, tested by inoculation of mice, reduces to 1% after 90 days of dapsone treatment [28]. However, its extensive use as monotherapy for leprosy before the introduction of MDT, has contributed to the development of dapsone resistant leprosy strains.

In the group of fluoroquinolones, **moxifloxacin** has the best bactericidal activity against *M. leprae* in a murine model [23]. It has a plasma half-life of around 12 h. The fluoroquinolones inhibit bacterial DNA synthesis by targeting the enzymatic activities of DNA gyrase, necessary for DNA replication. The bactericidal efficacy of moxifloxacin against *M. leprae* in the mouse footpad model was 92.1% [23]. Moxifloxacin is not routinely used as a drug for leprosy patients, but in a clinical proof-of-concept trial in eight multibacillary leprosy patients, moxifloxacin proved highly effective. In the trial patients, a single 400 mg dose of moxifloxacin resulted in significant killing (*P* ≤ 0.006) of *M. leprae*, ranging from 82 to 99%, with a mean of 91% [29]. Skin lesions improved rapidly; subtle improvement in some patients was observed after a single 400 mg dose of moxifloxacin. In all eight patients definite improvement was observed with an additional week of daily moxifloxacin (day 14). Moxifloxacin is currently used for treatment of leprosy in a clinical trial in India (Kar, personal communication, November 2016).

Unlike some other fluoroquinolones, moxifloxacin demonstrated to have low propensity for causing phototoxic reactions in animal studies. But other findings in safety studies in animals (e.g. arthrotoxicity in juvenile animals and CNS toxicity) that have led to restrictions in the use of quinolones in general, have also been observed with moxifloxacin. Similar to other fluoroquinolones, moxifloxacin has been shown to cause lesions in the cartilage of the weight-bearing joints of immature animals. It should therefore not be used in children, or pregnant women unless the benefits outweigh the risks. Even though, there is lack of correlation between findings in juvenile animals and those in children [30]. Preclinical studies, in which high doses of moxifloxacin were used, demonstrated the potential to induce convulsions. Caution is required with patients having an increased risk for tachyarrhythmia because it induces QT interval prolongation [31]. However, a meta-analysis done to determine its effectiveness and safety for tuberculosis treatment concluded that the combination of moxifloxacin with the recommended regimen for the treatment of TB, with a daily dose of 400 mg given for at least 2 months, does not cause additional adverse events [32].

The working mechanism of **ofloxacin** is similar to that of moxifloxacin, but the bactericidal effect proved to be much less than moxifloxacin in the mouse footpad model (60.2% vs. 92.1%) and less adverse effects were observed [23]. The plasma half-life of around 6 h is much shorter than that of moxifloxacin (around 12 h) and therefore the duration of the effect is also much shorter [33, 34].

**Minocycline** is a semisynthetic tetracycline. Its bactericidal activity was shown in a trial where a single 200 mg dose of minocycline was given to eight lepromatous patients. This decreased the number of patients with viable *M. leprae*, as demonstrated in mouse footpads (six of the eight patients) [35]. However, the bactericidal effectiveness of minocycline against *M. leprae* is less than that of rifampicin [36]. As with other tetracyclines, it is contraindicated in children because it leads to discoloration of developing teeth, and may cause hypersensitivity (DRESS syndrome) in some patients [37, 38]*.*

The bactericidal effect of **clarithromycin** for *M. leprae* was 74.9% in the mouse footpad model [23]. Clinical trials indicated that it is rapidly bactericidal for *M. leprae* in humans [39]. It is not routinely used in the treatment of leprosy, but it can safely be given to children. It was used as a prophylactic drug for leprosy in a trial in Indonesia conducted among healthy elementary school children in high-endemic areas in East Java province (Prakoeswa, personal communication, November 2016). An enhanced chemoprophylaxis regimen was given to children with high antibody titres (anti-PGL IgM) for leprosy: rifampicin 300 mg/day and clarithromycin 250 mg/day for the first ten days, followed by three months of intermittent two-weekly doses of rifampicin 300 mg and clarithromycin 250 mg. During the 5-year follow-up period the children were screened annually for signs/symptoms of leprosy and blood was taken to measure anti PGL-1 IgM titres. Although no control group was included in the study and a substantial proportion of the children was lost to follow-up, several important observations were made. Rifampicin and clarithromycin were well tolerated, no major adverse events were seen, none of the children was diagnosed with leprosy during the follow-up period, and 87% of the children had a decrease in the anti PGL-1 IgM titres.

### Qualifications of an optimal enhanced chemoprophylaxis regimen

An enhanced chemoprophylaxis regimen should meet several criteria. It should be highly effective, much more than the current SDR regimen, safe without substantial side effects, acceptable to people who are not ill, available, affordable, feasible to administer on a large scale, and should not induce drug resistance in leprosy or TB bacteria. Safety, effectiveness and affordability are commonly used to select chemoprophylaxis regimens, drug resistance is to be avoided in all use of antibiotics, the other criteria are more specific for the use of chemoprophylaxis for leprosy [40].

The first criterion is **effectiveness**. The new regimen should be sufficiently bactericidal to treat people harbouring higher numbers of *M. leprae* likely to be present in those with subclinical infection. Potentially, it should be capable of curing early MB leprosy. Given that the current SDR PEP regimen is 60% effective, the new regimen should be substantially more effective to be worthwhile, e.g. offering a protective effectiveness of 80 to 90%. For an increased preventive effect, a long-acting antibiotic and/or repeated administration is necessary. Repeating doses have a greater bactericidal effect than a single dose.

The second criterion is **safety**. The regimen will be given to healthy individuals as a preventive measure. People may be reluctant to take multiple drugs or multiple doses when they do not feel sick. It is therefore of greatest importance to avoid any adverse event to the extent possible. Contraindications for any of the drugs in the regimen should be checked carefully, before providing the regimen.

The third criterion is **acceptability**. The regimen should be easily acceptable for healthy contacts, because they are not motivated to take the drugs by being ill. The tablets or capsules should be easy to swallow and for smaller children the availability of the drug as a syrup would be preferred. Also, the schedule for the intake of the drugs should be simple enough to adhere to and the number of repeating doses should be limited. Furthermore, the regimen should ideally not induce any side effects, such as nausea, dizziness, headaches or skin discoloration.

The fourth criterion is **availability** of the drug. The drug should be available in the countries where the PEP++ research project is taking place at an **affordable** rate (fifth criterion).

The sixth criterion, **feasibility** to administer the regimen on a large scale is a combination of the above criteria, but also encompasses logistical aspects. It should be possible to widely distribute the drugs in adequate amounts. Storage requirements for the drugs should be simple and the shelf life sufficiently long.

The seventh criterion is that the prophylaxis should **not induce drug resistance** in *M. leprae* or *M. tuberculosis*. The probability of emergence of resistance depends on the mycobacterial load, the potency of drugs (pharmacodynamics) and the number of drugs (less in combinations of ≥2 drugs). It is known that repeated doses of one drug will increase the risk of the development of resistance. To prevent this, a combination of drugs is preferred when giving multiple doses [41]. Antimicrobial resistance is well known in leprosy. Rifampicin resistance was first described in 1976 and fluoroquinolones resistance in 1997 [42, 43]*.* The antimicrobial resistance proportion was estimated to be 8%, in a first prospective open survey conducted by a WHO surveillance network in the period 2009–2015 [44].

Specific interventions to address concerns about resistance, such as surveillance, will be established during the PEP++ research project. Additional logistical considerations must be taken into account in the development of the final field protocol. These considerations are beyond the scope of this report and will be detailed in a future publication.

### Possible drug combinations for enhanced chemoprophylaxis

Theoretically, one or two months of MDT treatment could be used as prophylactic treatment. Two months of MDT or one month of MDT ending with a monthly dose of rifampicin would appear to be a feasible regimen. The efficacy of MDT is known, it uses the same drugs for adults and children, it is available and it is accepted in all leprosy endemic counties. However, if standard MDT is given as preventive treatment, contacts and community members may perceive this as leprosy treatment and think that people taking this medication are leprosy patients. Moreover, on the basis of safety considerations, dapsone should not be included in the regimen since it occasionally causes a potentially fatal hypersensitivity syndrome [45], while its bacteriostatic properties are unlikely to have an additional beneficial effect. Clofazimine is not preferred because of the late bactericidal activity and the discoloration of the skin it causes. This is not easily accepted by people, as it is associated with being on treatment for leprosy.

ROM has been used to treat single lesion leprosy. Compared to rifampicin, ofloxacin and minocycline have been shown to be less bactericidal in mouse models [46, 47]. ROM is no more bactericidal than rifampicin alone [46]. A combination of rifampicin with a more powerful bactericidal drug would therefore be preferred for optimal efficacy of a chemoprophylactic regimen.

Rifampicin remains a good candidate as component of the enhanced regimen, although its short half-life is a limitation. The wide use of single-dose rifampicin in the LPEP project has shown to be safe (no serious adverse events have been reported to date), acceptable, available and affordable [17]. The Expert Meeting advised to combine rifampicin with moxifloxacin, the most bactericidal of the possible drugs for an enhanced regimen, with a longer half-life and good pharmacodynamic properties. It was agreed that the combination is likely to increase the protective effect of the chemoprophylaxis substantially, especially when repeated doses are given. Importantly, combining two antibiotics would reduce the likelihood of inducing resistance [48]. Fluoroquinolones in general and moxifloxacin specifically have been used as chemoprophylaxis for TB, also in children; no serious adverse events were reported [49–51]. However, fluoroquinolones are known to have potentially toxic side effects, especially in children, which need to be considered when they are used as prophylaxis instead of treatment of disease. The Expert Meeting therefore advised to replace moxifloxacin by clarithromycin when giving the chemoprophylaxis to children (< 16 years) and adults with a contraindication for moxifloxacin. Giving the combination of rifampicin and clarithromycin to all contacts, children as well as adults, is not preferred, because of the stronger bactericidal effect of moxifloxacin. Both rifampicin and clarithromycin can be given to children in syrup form.

To determine the frequency and duration of the use of the regimen, the Expert Meeting had the following considerations:

When rifampicin was introduced as component of MDT treatment, a monthly dose was recommended in view of the good therapeutic efficacy and the fact that this was better tolerated than a weekly dose and the lower costs compared to daily or weekly treatment [52]. The Expert Meeting strongly advised that the enhanced regimen should also be given supervised to ensure compliance and repeatedly to increase the bactericidal effect. From a logistical point of view, a regimen that will be given daily for seven days is difficult to administer as an observed dose. An interval of one week or less is likely to cause more side effects [53, 54]. A monthly dose is therefore preferred and more feasible as it is easier to be supervised. Giving three doses instead of one single dose was advised to increase the probability of killing more bacilli in an active metabolic state.

The contraindications used for SDR (pregnancy, liver or renal disorders, signs or symptoms of leprosy, signs or symptoms of TB, known allergy to rifampicin) would also apply to the enhanced regimen [16]. In addition people with known cardiac or neurological disease, especially people suffering from seizures should be given clarithromycin instead of moxifloxacin.

## Conclusion

The Expert Meeting concluded that an enhanced prophylactic regimen against leprosy would comprise multiple doses of rifampicin and moxifloxacin, since this would combine the two most bactericidal drugs available. Because moxifloxacin is not recommended in children rifampicin plus clarithromycin was considered the preferred regimen for child contacts and also for adults with moxifloxacin-specific contraindications.

For the PEP++ intervention study, the Expert Meeting proposed an enhanced regimen comprising three doses of rifampicin 600 mg (weight adjusted when given to children) plus moxifloxacin 400 mg given at four-weekly intervals (day 1, day 29 and 57) over 8 weeks. For children and for adults with contraindications for moxifloxacin, moxifloxacin should be replaced by clarithromycin 300 mg (weight adjusted).

## Abbreviations

EMA: European Medicines Agency
FDA: Food and Drug Administration
LPEP: leprosy post-exposure prophylaxis
M. (leprae/tuberculosis): mycobacterium
MB: multibacillary
MDT: multi drug treatment
NLR: Netherlands Leprosy Relief
PB: paucibacillary
PEP: post-exposure prophylaxis
PEP++: post-exposure prophylaxis enhanced regimen
SDR: single dose rifampicin
TB: tuberculosis
